# Development of Plug Joint with Polymer-Modified Rubber Asphalt as Filling Material

**DOI:** 10.3390/polym15214256

**Published:** 2023-10-29

**Authors:** Kyung-Nam Kim, Yeong-Min Kim, Tri Ho Minh Le

**Affiliations:** 1 Korea Expressway Corporation Research Institute Pavement Research Division, Dongtansunhwan-daero 17-gil, Hwaseong-si 18489, Gyeonggi-do, Republic of Korea; kkn248@ex.co.kr; 2 Department of Highway & Transportation Research, Korea Institute of Civil Engineering and Building Technology, 283 Goyangdae-ro, Ilsanseo-gu, Goyang-si 10223, Gyeonggi-do, Republic of Korea; 3 Faculty of Civil Engineering, Nguyen Tat Thanh University, 300A Nguyen Tat Thanh Street, District 4, Ho Chi Minh City 70000, Vietnam; lhmtri@ntt.edu.vn

**Keywords:** asphalt plug joint, polymer-modified rubber, asphalt pavement, bridge deck pavement, self-leveling

## Abstract

Rising traffic volume, heavy loads, and construction activities have raised concerns about expansion joint device damage. This study focuses on developing an innovative expansion joint using polymer-modified rubber asphalt as the filling material to enhance its service life. Styrene–butadiene–styrene (SBS) emerged as a suitable modifier for rubber-modified asphalt, significantly improving elasticity and adhesion. Through the strategic combination of 3- and 2-block linear SBS, the elasticity and adhesion properties were significantly improved, resulting in the formulation of a well-suited polymer-modified rubber asphalt binder. The developed asphalt binder exhibits impressive elastic recovery (61.1% to 66.1%), surpassing commercial products, with enhanced constructability and workability (15% to 21% viscosity reduction). The carefully engineered mastic asphalt mixture showcases self-leveling characteristics at a moderate 210 °C, addressing historical constructability challenges. Settlement is 40% less than traditional hot mix asphalt for surface layers, with improved moisture and stripping resistance, enhancing existing asphalt plug joint durability and workability. Collectively, this novel mixture, comprising polymer-modified rubber and mastic asphalt, showcases the potential to enhance the durability of existing asphalt plug joints while ensuring superior constructability and workability.

## 1. Introduction

Expansion joints serve a critical function in accommodating the dynamic movement and rotation of a bridge’s upper structure, ensuring the integrity and safety of the entire bridge system [1]. Despite their often-underestimated role, these components play a pivotal role in assessing the overall condition of a bridge [2]. Damage to expansion joint devices not only compromises the functionality of the bridge but can also lead to reduced ride quality, traffic accidents, and disruptions [3]. Consequently, ongoing maintenance efforts are essential to preserve their performance [4]. Unfortunately, the service life of an expansion joint tends to be shorter than that of the bridge itself, and the repair or replacement of these joints constitutes a significant portion of a bridge’s total maintenance cost [5,6,7,8].

**Figure 1:**
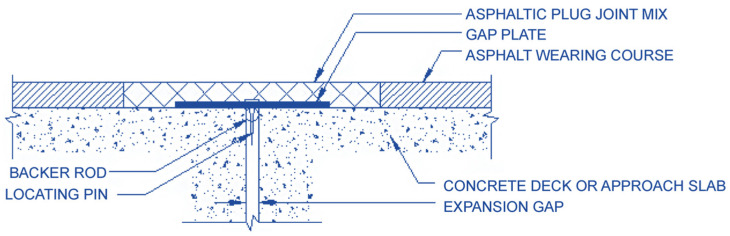
Schematic of an asphalt plug joint cross-section.

In response to these challenges, there has been a growing interest in the development of buried joints designed to enhance driving comfort and expedite construction [9]. Buried joints, which are engineered to absorb the expansion, contraction, and structural impacts of the bridge superstructure, offer an effective solution. As illustrated in Figure 1 based on a concept from Mogawer and Austerman, 2004 [10], this construction method involves the placement of a backing material and steel plate within an oil-filled chamber, combined with an asphalt mixture composed of aggregates and bitumen. This innovative approach improves stress absorption and ride quality, ultimately enhancing the performance of expansion joints.

One of the key requirements for the success of these buried joints is the sealant material used to absorb stresses in bridges and various concrete structures, including underpasses, tunnels, and box-type culverts [11]. Notably, an elastic restoring force within the range of 40–70% is critical to ensuring optimal drivability by minimizing deformation caused by repetitive traffic loads. Additionally, the sealant must exhibit exceptional water resistance to protect against the corrosion of steel components. In practice, polymer-modified rubber asphalt has emerged as the preferred filling material for these buried joints (commonly referred to as asphalt plug joints or APJs) [12,13,14,15]. The choice of polymer-modified rubber asphalt is underpinned by its impressive attributes [16], including superior elasticity, adhesion, and water resistance [17,18,19,20]. Moreover, this material’s versatility extends to applications in roof waterproofing and as a sealant. Its widespread adoption worldwide is a testament to its excellent processability and ease of construction [15].

Additionally, recent research in the field of SBS (styrene–butadiene–styrene) polymer-modified asphalt has seen significant advancements aimed at enhancing the performance and durability of asphalt materials [21,22,23]. These studies have focused on various aspects, including the selection of optimal SBS modifiers to improve elasticity and adhesion [24], the development of innovative polymer-modified asphalt binders [25], and the exploration of novel applications in construction and pavement engineering [20,26]. Researchers have been actively investigating the effects of different SBS types and blending ratios on asphalt properties, with the aim of achieving superior elasticity and resistance to deformation [27,28]. Advancements in the characterization and testing of SBS-modified asphalt have allowed for a more comprehensive evaluation of its performance under varying conditions, further refining its suitability for diverse applications [29,30]. Recent research in this area has paved the way for the development of high-quality, long-lasting asphalt materials with improved workability and resistance to factors such as moisture and fatigue, making them integral to the construction and maintenance of modern infrastructure [31,31]. Furthermore, in recent studies [32], significant advancements have been made in understanding the effects of weather aging on modified asphalt with rubber–polyethylene composites and the investigation of storage stability and rheological properties of environmentally friendly devulcanized rubber modified asphalt, respectively. These findings provide valuable insights into the durability and performance of modified asphalt materials under various conditions. For examples, Ma et al.’s research sheds light on the impact of weather aging on rubber–polyethylene composites [33], while Zhou et al.’s work contributes to a better understanding of the storage stability and rheological characteristics of devulcanized rubber-modified asphalt [34]. It becomes evident that such factors are crucial in assessing the durability and ultimately the extended service life of asphalt pavement joints. Typically, the service life of an APJ ranges from 6 to 7 years, contingent upon its ability to mitigate shocks, provide a comfortable driving experience, and resist water ingress. Unfortunately, premature failure can manifest in as little as 6 months under certain conditions [9,35]. Various forms of damage, including plastic deformation and cracking, may occur due to factors such as vehicle loads, temperature fluctuations, humidity, and oxygen levels, all of which impact the APJ application zone [10,36,37,38,39,40,41]. Consequently, strengthening APJ materials becomes imperative to ensure the long-term performance of expansion joint devices.

Recent research in the field of expansion joints for bridge infrastructure has highlighted several critical issues and gaps that demand attention. These gaps primarily revolve around the need for more durable, resilient, and cost-effective materials to extend the service life of expansion joints, given the increasing traffic loads and environmental challenges. Existing materials, though widely used, often fall short in terms of elasticity, adhesion, and resistance to plastic deformation and moisture. The current literature reflects a gap in exploring innovative materials and approaches to address these limitations comprehensively. Additionally, there is a lack of emphasis on improving workability and constructability during expansion joint construction, which is crucial for efficient and cost-effective bridge maintenance. These research gaps underscore the urgent need for novel solutions that can enhance the performance, durability, and sustainability of expansion joints in bridge infrastructure.

**Figure 2:**
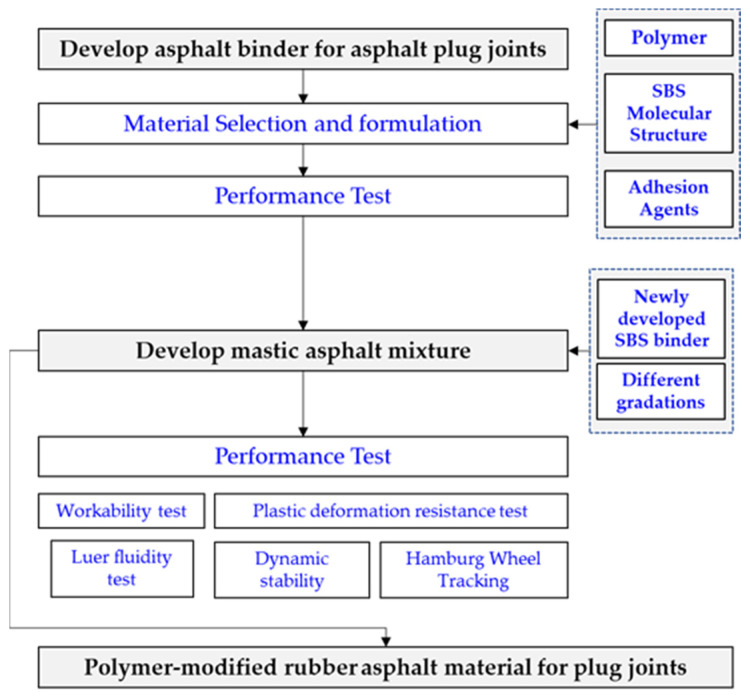
Flowchart for developing asphalt plug joint with polymer-modified rubber as filling material.

Motivated by these challenges and driven by the need to enhance the service life of expansion joints, this study embarks on the development of a novel expansion joint employing polymer-modified rubber asphalt as the filling material. The novelty of this research lies in its innovative approach to addressing the longstanding challenges of expansion joints in bridge infrastructure. By developing expansion joints with polymer-modified rubber asphalt filling materials, this study breaks new ground in the field of civil engineering and materials science. The selection of styrene–butadiene–styrene (SBS) as a modifier for rubber-modified asphalt, the meticulous formulation of a polymer-modified rubber asphalt binder, and the exploration of mastic asphalt mixtures represent pioneering steps towards achieving expansion joints with exceptional elasticity, adhesion, and durability. Furthermore, the research introduces a unique focus on improving workability and constructability through particle size adjustments in the mastic asphalt mixture. The application of self-leveling properties at 210 °C represents a significant advancement in construction practices, enhancing efficiency and reducing labor costs. Overall, this research offers a fresh perspective on the enhancement of expansion joint materials, paving the way for safer and longer-lasting bridges, thus making a substantial contribution to the field of civil engineering and infrastructure sustainability. The research journey, illustrated in Figure 2, encompasses the formulation of a polymer-modified rubber asphalt binder tailored for expansion joints, marking a significant step toward improving the durability and performance of these crucial bridge components.

## 2. Materials and Methods

### 2.1. Expansion Joint Filling Material

In this research, all materials used in this research were provided by the Korean Asphalt Company, situated in Seoul, Republic of Korea. The APJ fills the expansion joints with an asphalt mixture and ensures flatness by seamlessly connecting to the pavement. These joints are used to resolve the expansion and contraction occurring in a structure by absorbing it from the expansion joint material [42]. One of the advantages of APJ is its good drivability, as almost no shock, vibration, or noise exists. Additionally, it is completely waterproof; thus, the APJ is easy to install and maintain, and it is relatively inexpensive [15]. Generally, APJs are applied to a maximum allowable extension of 50 mm, and polymer-modified asphalt is typically used [12].

**Figure 3:**
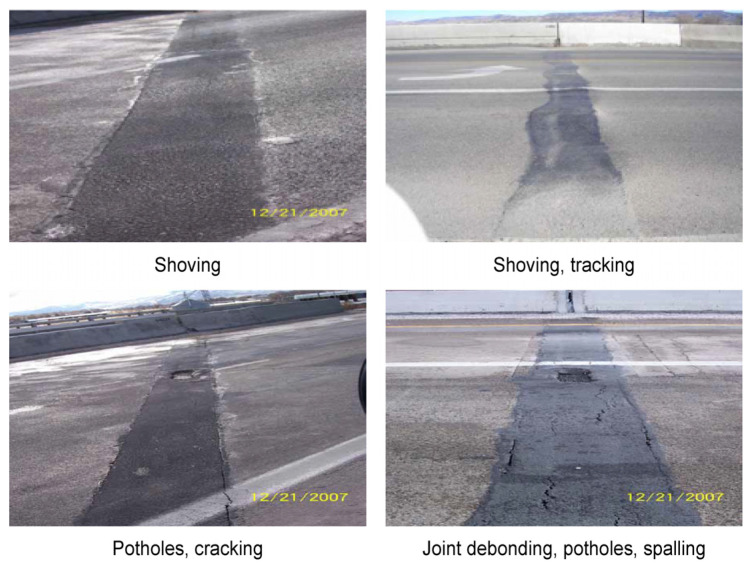
Damage types of asphalt plug joints.

**Figure 4:**
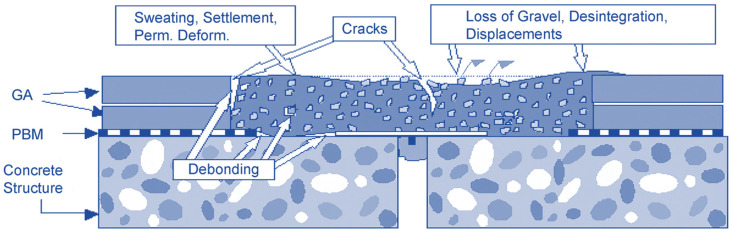
Typical distress types of asphalt plug joints.

Because the APJ constitutes an expansion joint with an asphalt mixture, the ductility increases as the temperature increases, and the brittleness increases as the temperature decreases. These characteristics increase the risk of plastic deformation when the traffic volume is large at high temperatures. When the traffic volume is small, swelling occurs, and the pavement can be damaged. Conversely, at low temperatures, the material becomes brittle and develops cracks, leading to leakage and desorption of the pavement [14]. Therefore, the asphalt material used for APJ must have excellent temperature sensitivity and physical properties to withstand the shrinkage and expansion of a structure. Figure 3 shows the typical fracture shape of an APJ, and Figure 4 presents a schematic of the fracture shape based on the original concept from Mogawer and Austerman, 2004 [10].

Expansion joints are more vulnerable to breakage than the surrounding structures and pavement. Thus, damage frequently occurs in the expansion joints. In addition, the damage initiated by the expansion joint generally increases the impact coefficient of a passing vehicle. Consequently, the damage factor and damage speed are increased, which can cause severe damage to the bridge [13]. APJs are soft at high temperatures owing to the material characteristics of the asphalt binder. When the temperature is low, they are sensitive to temperature and become brittle. In high-temperature environments, rutting, heaving, and delamination may occur. In contrast, at low temperatures, problems such as spalling, potholes, joint damage, and dropout may occur [43]. In the US, ASTM D 6297 (2020) is the quality standard for asphalt binders used in APJs [41]. However, in Korea, no similar standards exist other than the quality standards for asphalt-based joint materials. Because the joint material is injected into a narrow gap, fluidity is important, and no standard item related to the load-bearing capacity exists concerning vehicle traffic. Accordingly, for APJs, a hard asphalt binder must be applied, with a sufficient bearing capacity to resist the load of vehicles passing over the expansion joint. 

### 2.2. Developed Asphalt Binder for APJ

#### 2.2.1. Polymer Selection

The Korean Asphalt Company, located in Seoul, Republic of Korea, supplied all the materials utilized in this study. Due to its production and construction characteristics, the APJ should have an affinity for asphalt, a high softening point, good elastic recovery, and a simple manufacturing process. Table 1 lists the physical properties of the materials that improve the elasticity of the asphalt material. The table analysis indicates that the polymer material based on styrene–butadiene–styrene (SBS) is the most suitable. 

Table 1 provides an overview of the physical properties of different elastic force-imparting polymers, namely SBS, SBR (Styrene Butadiene Rubber), BR (Butadiene Rubber), and CRM (Crumb Rubber Modifier). These properties include the vulcanization process requirement, softening point, elastic resilience, process suitability, and asphalt compatibility. SBS stands out for not requiring vulcanization, having a high softening point, excellent elastic resilience, and process suitability, making it a preferred choice for various applications, including asphalt modification. In contrast, SBR necessitates vulcanization, has a lower softening point, and moderate elastic resilience, while BR and CRM exhibit characteristics that may require equipment or process adjustments for optimal performance in specific applications. This table serves as a valuable reference for selecting the most suitable polymer based on the desired properties for a given application.

#### 2.2.2. SBS Molecular Structure Selection

**Figure 5:**
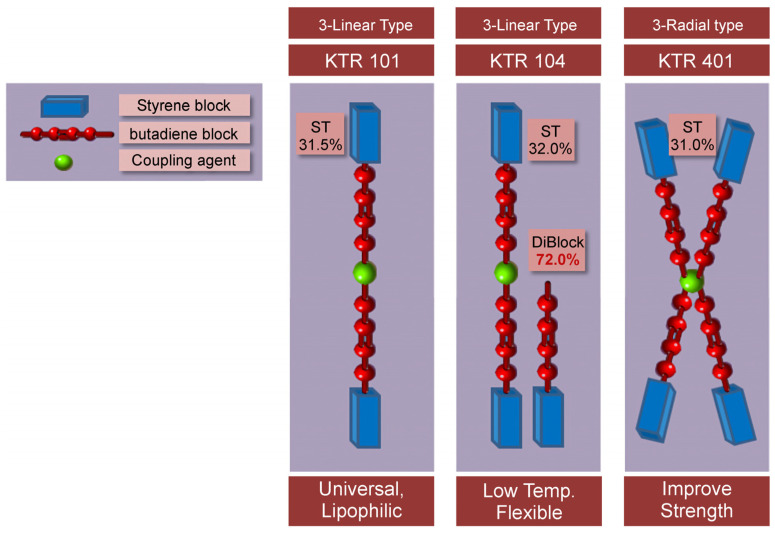
Schematic of the styrene–butadiene–styrene molecular structure.

Because SBS has different properties depending on its molecular structure, this material must be selected with a structure suitable for the APJ. Figure 5 shows a schematic of the SBS molecular structure. In addition, Table 2 lists the physical properties of the asphalt binder to which SBS is applied. In this study, APJ materials were developed with a high softening point, elongation, and low viscosity. A mixture of 2- and 3-block linear SBS was selected. 

Table 2 presents a comparative analysis of physical properties among different SBS asphalt binder materials, categorized into two distinct structural types: 3-Linear and 3-Radial. This table outlines key properties, including softening point, ductility at 25 °C, penetration at 25 °C (using 100 g for 5 s), viscosity at 135 °C, and elastic recovery at 25 °C. The data reveals notable variations in these properties based on the SBS structural type. The 3-Linear type exhibits a softening point of 108.3 °C, higher ductility (140 cm), and higher penetration (23.4 dm), suggesting improved flexibility and workability. In contrast, the 3-Radial type has a higher softening point (114.2 °C) but shows lower ductility (75 cm) and penetration (13.5 dm). Additionally, the 3-Linear type demonstrates lower viscosity at 135 °C (5600 cPs) compared to the 3-Radial type (11,700 cPs). These findings provide valuable insights for selecting the appropriate SBS asphalt binder material based on specific project requirements and performance expectations.

Continuing with Table 2, it is evident that the choice between 3-Linear and 3-Radial SBS asphalt binder materials significantly impacts the physical properties of the final product. The 3-Linear type showcases a higher elastic recovery rate of 95% at 25 °C, indicating its ability to regain its original shape after deformation, which can be advantageous for applications requiring resilience. On the other hand, the 3-Radial type exhibits a slightly lower elastic recovery rate of 94% at the same temperature.

These distinctive characteristics have practical implications for various asphalt-related applications. For instance, the 3-Linear SBS structure’s higher ductility, penetration, and lower viscosity make it well-suited for scenarios where improved flexibility and ease of application are paramount. Conversely, the 3-Radial type, with its higher softening point and comparable elastic recovery, might be preferred when higher temperature resistance is required. Engineers, construction professionals, and manufacturers can use these data to make informed decisions when selecting SBS asphalt binder materials tailored to specific project demands, optimizing performance and longevity in various environmental conditions and applications.

#### 2.2.3. Selection of the Adhesion Agents

To prevent damage and drop-off of the joint, the APJ must adhere to dissimilar materials, such as asphalt and cement concretes. Typically, petroleum resin is used to strengthen the adhesion and cohesion of heterogeneous materials. In this study, three types of petroleum resins were reviewed. Their physical properties are summarized in Table 3.

In Table 3, the research aims to explore the physical properties of different petroleum resin types, namely Type A, Type B, and Type C, each represented by distinct grades. These grades, G140, G120, and G1100, exhibit varying characteristics that are crucial in determining their applicability in asphalt-related industries. Type A, represented by grade G140, stands out with its high softening point of 145 °C and excellent compatibility with asphalt. This makes it a prime choice for applications demanding superior temperature resistance and asphalt compatibility. Type B, represented by grade G120, also offers excellent asphalt compatibility but at a slightly lower softening point of 120 °C, making it suitable for scenarios requiring a balance between performance and cost-effectiveness. Type C, with grade G1100, possesses a softer characteristic with a 100 °C softening point and exhibits moderate compatibility with asphalt. This makes it a viable option for applications where a lower softening point is acceptable, and a cost-effective solution is desired. The availability of these petroleum resin types with distinct properties empowers professionals in the asphalt industry to select the most appropriate material for their specific project requirements, ensuring optimal performance and durability.

The physical properties of the asphalt binder (e.g., softening point and viscosity) depend on the petroleum resin used. Therefore, the physical properties of the material after stirring with the treated asphalt binder were examined. Table 4 lists the physical properties of the petroleum resins used. The softening point, stripping resistance, and phase-segregation characteristics of different materials were compared by preparing specimens using a high-speed shear mixer with a ratio of 90% straight asphalt (AP-5) and 10% petroleum resin (by weight).

Table 4 delves into the physical properties of asphalt binders enriched with different grades of petroleum resin, specifically Type A with grade G140, Type B with grade G120, and Type C with grade G1100. These modified asphalt binders exhibit distinct characteristics that can significantly impact their performance in various applications. Type A, represented by grade G140, showcases a softening point of 98.3 °C, indicating its ability to maintain stability at elevated temperatures. It also exhibits good stripping resistance at 90 °C, with a value of 2.21 kgf/cm, indicating its resistance to moisture-induced damage. Moreover, it displays minimal phase segregation, with a Δ°C of 2.2 at 163 °C over 48 h, highlighting its stability during prolonged heating. Type B, represented by grade G120, boasts a softening point of 92.7 °C, making it suitable for applications requiring moderate temperature resistance. It also demonstrates good stripping resistance (2.32 kgf/cm) and minimal phase segregation (Δ°C of 2.3), contributing to its overall reliability. Type C, featuring grade G1100, has a softening point of 85.4 °C, making it a cost-effective choice for applications where lower-temperature resistance is acceptable. However, it exhibits slightly lower stripping resistance (2.22 kgf/cm) and significantly higher phase segregation (Δ°C of 13.1), which may impact its performance in specific scenarios. These findings empower professionals in the asphalt industry to select the most suitable asphalt binder with petroleum resin additives based on their project’s unique requirements, ensuring durability and optimal performance.

In the stripping resistance test, the three types of mixtures considered in this study exhibit similar characteristics. The softening point and compatibility with asphalt materials confirm that Type A petroleum resin was most suitable.

#### 2.2.4. Asphalt Binder Formulation

The primary formulation of APJ was designed through a material review. The materials considered for the primary formulation were 2-block linear SBS, 3-block linear SBS, and G140-grade petroleum resin. The formulation compositions are summarized in Table 5.

Table 5 provides essential insights into the mixed proportion of the filling asphalt plug joint material, specifically designed for the development of an expansion joint. This joint material is categorized into two divisions, referred to as Type 1 and Type 2, each with its distinct composition ratios:

For Type 1 joint material, the composition includes PG 64-22 asphalt as the primary component, constituting 75% of the mixture. Additionally, it incorporates 10% SBS of both 101 and 104 grades, enhancing its elastic properties. Furthermore, Type 1 includes 10% G140 petroleum resin and 5% GS150 oil, contributing to its durability and adhesion characteristics. Notably, no mineral (CaCO_3_) is included in Type 1.

Type 2 joint material also utilizes PG 64-22 asphalt as the base, comprising 75% of the mixture. However, it diverges by using 6% SBS 101 and 4% SBS 104, offering a slightly different blend of elastic properties compared to Type 1. Similar to Type 1, Type 2 includes 10% G140 petroleum resin and 5% GS150 oil. Notably, Type 2 also excludes mineral (CaCO_3_) from its composition.

These precise composition ratios enable the creation of specialized filling asphalt plug joint materials, catering to specific requirements and applications within the context of expansion joint development, ultimately contributing to enhanced road infrastructure performance and longevity.

The quality test results indicate that the ASTM D 6297 [41] quality standards for the softening point, penetration, and elastic recovery were satisfied, as summarized in Table 6. The developed product demonstrated softness comparable to that of commercial APJ products in the penetration test, with approximately 4–10 dm of penetration. However, in the case of Type 2, the softening point exceeded the reference value by ≥20 °C, and the elongation was twice the reference value approximately, improving the overall physical properties, such as the increase in softening point, decrease in viscosity, and increase in elasticity.

Table 6 presents a comprehensive analysis of the physical properties of the first developed expansion joint filling material, following ASTM standards. This material is divided into two distinct types, referred to as Type 1 and Type 2. Notably, both types exceed the specified requirements for several key properties. The softening point, crucial for application in various temperature conditions, surpasses the minimum requirement of 83 °C, with Type 1 and Type 2 exhibiting values of 108.3 °C and 103.4 °C, respectively. Ductility, essential for flexibility and durability, significantly exceeds the 400 mm minimum requirement, with Type 1 and Type 2 showcasing impressive values of 760 mm and 880 mm, respectively. Furthermore, both types satisfy the recommended application heating range of 182–199 °C. Although specific penetration and viscosity targets are not provided in the specifications, Type 1 and Type 2 demonstrate distinct penetration values of 27.8 dm and 33.9 dm, as well as viscosity values of 6691 cPs and 5675 cPs, respectively, at 135 °C.

These results illustrate the excellent performance and suitability of the first developed expansion joint filling material in meeting or exceeding industry standards. With superior properties such as high softening points, exceptional ductility, and adherence to recommended heating ranges, this material holds great promise for various construction and infrastructure applications, ensuring longevity and reliability in challenging environmental conditions.

In addition, material blending was performed to ensure economic feasibility while maintaining the physical properties of the primary design. The detailed composition of Type 3 was adjusted, and Type 4 was a combination of inorganic substances added to secure economic efficiency. As summarized in Table 7, the test results satisfied the ASTM D 6297 quality standards [41].

Table 7 provides an extensive overview of the physical properties of the final developed expansion joint filling material, following ASTM standards. This material is categorized into two distinct types, known as Type 3 and Type 4, and it exhibits excellent performance across various critical parameters. Both types of the material surpass the specified softening point requirement of 83 °C, with Type 3 and Type 4 displaying values of 102.5 °C and 104.5 °C, respectively. Tensile adhesion, an essential property for bonding and structural integrity, significantly exceeds the minimum requirement of >700%, with Type 3 and Type 4 achieving impressive values of 823% and 785%, respectively.

Additionally, the ductility of Type 3 and Type 4, essential for flexibility and durability, substantially exceeds the 400 mm minimum requirement, with Type 3 demonstrating a value of 760 mm and Type 4 showing 570 mm. The cone penetration values at 25 °C indicate good consistency for both types, with Type 3 and Type 4 exhibiting values of 23.3 and 18.1, respectively. Moreover, the material demonstrates excellent flow characteristics, resiliency, asphalt compatibility, and flexibility. These results underline the exceptional performance and suitability of the final developed expansion joint filling material for diverse construction and infrastructure applications, promising longevity and reliability under various environmental conditions.

Continuing with the analysis of Table 7, the material’s performance is further emphasized by its properties at lower temperatures. The cone penetration at −18 °C, a critical factor for materials used in cold climates, demonstrates excellent behavior for both Type 3 and Type 4, surpassing the specified requirement with values of 11.1 and 10.6, respectively.

The flow at 60 °C for both types is well below the maximum limit, with Type 3 and Type 4 showcasing values of 0.87 mm and 0.51 mm, respectively, indicating their stability under elevated temperatures. Resiliency, an important characteristic for materials that need to recover their shape after deformation, is well within the recommended range of 40–70%, with Type 3 and Type 4 achieving values of 66.1% and 61.1%, respectively.

The material also exhibits excellent asphalt compatibility, which is crucial for its successful integration into construction projects. Both Type 3 and Type 4 pass this test. Furthermore, the recommended application heating temperature range of 182–199 °C is met by both types, ensuring ease of use and proper application.

Lastly, the material’s penetration at 25 °C and elastic recovery further solidify its exceptional performance, with Type 3 and Type 4 showing values of 34.4, 20.4, 100, and 97, respectively. The viscosity at 135 °C, which affects the material’s handling and application, is well within acceptable limits, with Type 3 and Type 4 displaying values of 3200 and 4500 cPs, respectively. In conclusion, Table 7 underscores the outstanding quality and versatility of the final developed expansion joint filling material, making it a valuable choice for a wide range of construction and infrastructure projects.

### 2.3. Testing Methods

#### 2.3.1. Softening Point

Based on the ASTM D36 standard [44], in the softening point test, softening points of 102.5 and 104.5 °C were measured for Type 3 and Type 4 materials, respectively. These temperatures significantly exceed the quality standard temperature (83 °C) and softening points of commercial APJ products (88.5 °C), indicating excellent high-temperature performance. 

#### 2.3.2. Freshness and Flow

The elongation exhibited a high value that significantly exceeds the ASTM D 6297 standard (400 mm). This is because the viscosity was reduced using a low-viscosity 2-block linear SBS and oil. Moreover, 2-block linear SBS is considered advantageous in securing flexibility and elasticity compared with commercial APJ products that barely meet the quality standards. In the flowability evaluation, 3-block linear SBS and 2-block linear SBS maintain the SBS block formation at a test temperature of 60 °C, which satisfies the test criteria. Thus, the flow-down phenomenon in high-temperature environments can be avoided.

#### 2.3.3. Elastic Recovery Rate

**Figure 6:**
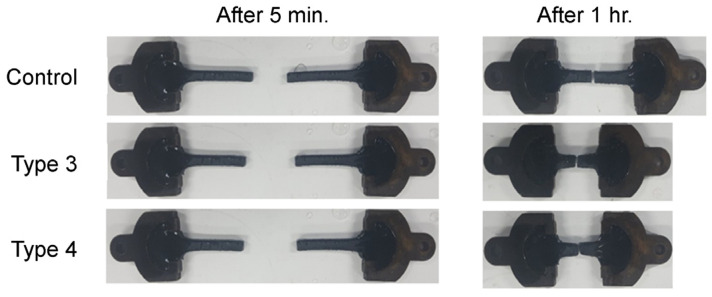
Specimen after the elastic recovery rate test.

In the elastic recovery rate test, the resulting rate for an overseas commercial product was 44.5%, close to 40% of the lower limit of the quality standard. The results for Type 3 and Type 4 materials (66.1% and 61.1%, respectively) are close to the upper limit, indicating that these materials have excellent elastic recovery. In addition, as shown in Figure 6, the recovery rate of the specimen after 1 h of the test is 97–100% for the developed material, whereas for overseas commercial products, more than 20% permanent deformation is measured.

#### 2.3.4. Viscosity

According to the viscosity test, the developed material had a viscosity of 3200–4500 cPs at 135 °C, representing 15–21% of that of overseas commercial products (20,000 cPs), indicating superior workability and stability. 

#### 2.3.5. Storage Stability Test 

The Storage Stability Test, conducted in this study, was implemented to assess the long-term performance and stability of different bitumen binders, namely Control, Type 3, and Type 4, as potential expansion joint filling materials. This test was executed following the ASTM-D7173 standard for determining the softening point of these binders [50]. Each binder sample underwent 48 h storage at a high temperature of 163 °C in an aluminum foil tube, simulating extreme conditions that the material might encounter in the field. After the storage period, the softening point of the top and bottom sections of the binder was measured. The difference in softening points between these two sections was used as a key indicator of storage stability. If this difference remained minimal, generally less than 2.5 °C, it indicated favorable storage stability. This method effectively evaluated the ability of the bitumen binders to maintain their properties and structural integrity when subjected to prolonged storage under high-temperature conditions.

#### 2.3.6. Frequency Sweep Test (FST)

The Frequency Sweep Test (FST) is a critical laboratory evaluation method for assessing the rheological properties of asphalt binders across a wide range of temperatures and time conditions. It involves the use of a dynamic shear rheometer (DSR) following the ASTM D7552-22 standard to measure the complex shear modulus (G*) and the phase angle (θ) [51]. These measurements help in characterizing how asphalt binders behave under different temperature scenarios, enabling predictions of their performance in various climate conditions. The FST’s unique feature is the creation of a dynamic shear modulus master curve, which combines time and temperature variables into a single curve for effective comparison and analysis of different asphalt binders. This method is crucial for optimizing asphalt binder formulations and selecting materials for road construction projects, ensuring the long-term durability of road pavements.

### 2.4. Mastic Asphalt

In this study, a modified rubber asphalt binder was developed as a filling material for expansion joints. The developed material has a higher elastic recovery rate and elongation than commercial APJ products. Moreover, the applicability of the developed APJ material obtained by applying the developed asphalt binder was verified considering a mastic asphalt mixture, generally used as a bridge pavement material.

Bridge pavements are largely divided into steel and concrete floor plates. For a steel deck bridge with large vibration and bending deformation, mastic asphalt is mainly applied because bending flowability and water resistance of the pavement are required. Despite its high cost, the mastic asphalt method has been introduced in Europe and Japan owing to its strong adhesion to the top plate and excellent durability. Recently, a mastic asphalt mixture has been applied to bridge pavements and general pavement sections to replace the waterproofing layer and suppress reflection cracks. Because compaction is unnecessary, mastic asphalt mixtures are also applied to narrow spaces, for example, for excavation and restoration. 

This study developed a mastic asphalt mixture with high resistance to cracking and plastic deformation and self-leveling characteristics owing to its excellent elastic recovery.

#### Material Development and Evaluation

The mastic asphalt mixture developed in this study is capable of self-compacting and self-leveling; consequently, improving its workability is the next focus. In addition, to use the mixture as a material for the surface layer, a performance test was performed. ASTM 6297 (standard specification of APJs for bridges) was used as the quality standard for the asphalt binder of the mastic asphalt mixture. As summarized in Table 8, the test results, such as softening point, tensile performance, and elongation, significantly exceed the quality standards. This indicates that the developed asphalt binder can be applied to mastic asphalt mixtures.

In general, Table 8 presents a comprehensive evaluation of the quality of the mastic asphalt binder. Notably, the test results consistently surpass the specified requirements, indicating a high-quality product ready for use in construction and infrastructure projects.

The softening point, a crucial parameter for asphalt materials, greatly exceeds the minimum requirement of 83 °C, with a test result of 102.5 °C. This indicates that the mastic asphalt binder can withstand elevated temperatures without softening, ensuring its durability in various environmental conditions.

Tensile adhesion, a measure of the binder’s ability to adhere to surfaces, is outstanding, with a test result of 823%, significantly exceeding the minimum requirement of 700%. This property is essential for the binder’s effectiveness in adhering to substrates and ensuring the longevity of constructed elements.

Ductility, another vital characteristic for asphalt materials, also surpasses the minimum requirement of 400 mm, with a test result of 760 mm at 25 °C. This indicates that the binder can withstand deformation without cracking or breaking, contributing to its suitability for flexible pavements.

The penetration at 25 °C is higher than expected, with a value of 34.4 dm. While penetration is typically not specified, this result indicates the material’s ability to accommodate traffic loads without becoming too soft or brittle.

Elastic recovery, an indicator of the material’s ability to return to its original shape after deformation, achieves a perfect score of 100% at 25 °C, emphasizing its resilience and suitability for various construction applications.

Lastly, the viscosity at 25 °C, which influences the material’s handling and application, meets the quality standards with a value of 3200 cPs. In summary, Table 8 confirms that the mastic asphalt binder is of exceptional quality, meeting or exceeding all relevant criteria, and is well-prepared for use in demanding construction projects.

**Figure 7:**
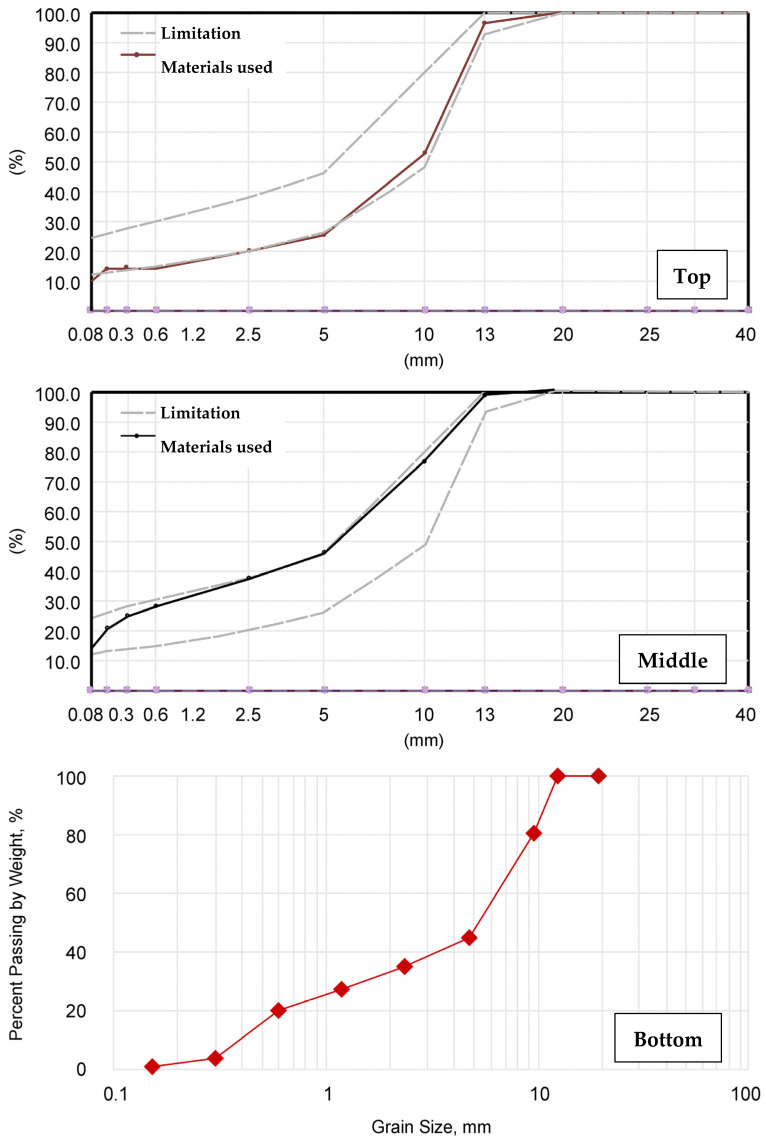
Aggregate particle sizes (rough gradation (**top**), soft gradation (**middle**), and workability gradation (**bottom**)).

The particle size of the mastic asphalt mixture and the capability for self-compacting and self-leveling were examined to ensure constructability. As shown in Figure 7, among the standard particle sizes of the mastic asphalt mixture, the rough gradation (RG) is close to the lower limit, and the soft gradation (SG) is close to the upper limit. Additionally, the fluidity test was performed for three different particle sizes with a high-fluidity cement concrete particle size (workability gradation, WG). 

## 3. Results and Discussions 

### 3.1. Binder Test Results

#### 3.1.1. Storage Stability Test Results Based on the Difference in the Softening Point

Table 9 presents the results of the storage stability tests for the developed expansion joint filling materials, including the Control, Type 3, and Type 4 formulations. These tests were conducted to assess the ability of these materials to maintain their softening points under elevated temperature conditions, simulating long-term storage. The softening point is a critical indicator of a material’s ability to withstand high temperatures without deforming or losing its structural integrity.

For the unaged samples, the Control material exhibited a softening point of 62.6 °C, while Type 3 and Type 4 showed slightly higher values of 65.2 °C and 64.8 °C, respectively. This indicates that the Control material had a lower initial softening point, suggesting that the addition of modifiers in Type 3 and Type 4 may enhance its high-temperature performance.

When assessing the top and bottom sections of the materials after the storage stability test, it was observed that Type 3 maintained the highest softening point in both sections. The softening point of the top section in Type 3 reached 65.0 °C, while the Control and Type 4 had values of 63.5 °C and 64.9 °C, respectively. In the bottom section, Type 3 also exhibited a higher softening point (65.1 °C) compared to the Control (62.9 °C) and Type 4 (64.4 °C).

The softening point difference, representing the change in softening point between the top and bottom sections after the storage stability test, provides insights into the material’s uniformity. Type 3 demonstrated the lowest softening point difference at 0.2 °C, indicating excellent uniformity and stability. The Control and Type 4 materials had slightly higher softening point differences of 0.9 °C and 0.5 °C, respectively.

These findings suggest that Type 3 exhibits superior storage stability, maintaining higher softening points and demonstrating less variation between the top and bottom sections, compared to the Control and Type 4 materials. This indicates that the formulation in Type 3 may offer improved long-term performance and resistance to temperature-induced changes, making it a promising candidate for expansion joint applications.

#### 3.1.2. Frequency Sweep Test Result

**Figure 8:**
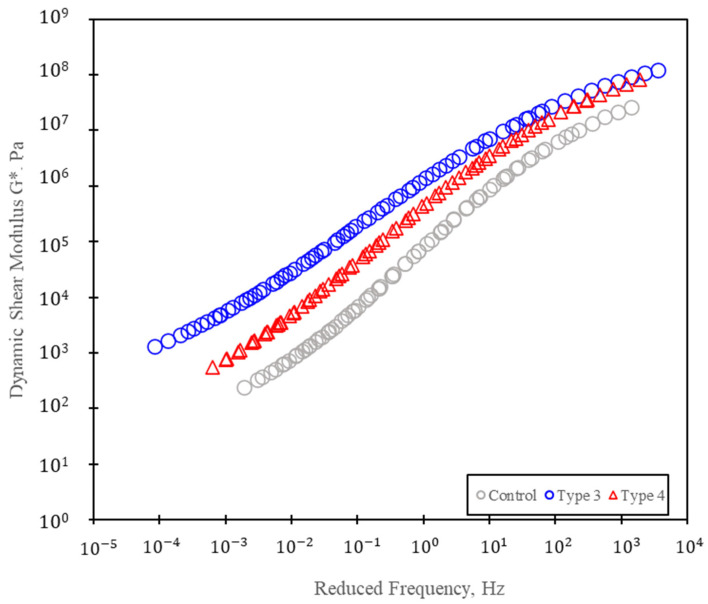
Frequency Sweep Test results.

The results of the Frequency Sweep Test (FST) conducted on the asphalt binders are presented in Table 7. This test was performed to evaluate the rheological properties of the Control, Type 3, and Type 4 asphalt binders over a range of temperatures and frequencies. The dynamic shear modulus (G*) values were measured at various temperatures, ranging from 13 °C to 90 °C, and at multiple angular frequencies as detailed in Figure 8. The master curve of the dynamic shear modulus was created using the FST results. As shown in Figure 8, the Control mixture exhibited the lowest G* values at all tested temperatures and frequencies. In contrast, the Type 3 binder consistently displayed the highest G* values across the entire temperature and frequency range, indicating superior stiffness. Type 4 demonstrated intermediate G* values between Type 3 and the Control. These results suggest that Type 3 is the stiffest binder, followed by Type 4, with the Control binder being the least stiff among the tested mixtures.

### 3.2. Workability Evaluation

**Figure 9:**
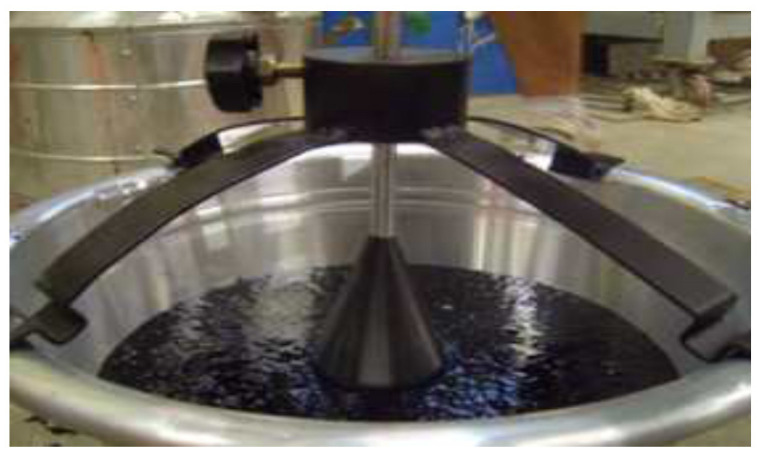
Luer fluidity test.

Based on the suggestion from Kim et al. [52], the Luer fluidity test was performed to evaluate the workability of the mastic asphalt. As shown in Figure 9, the test involves a 1 kg weight free-falling into a mixture heated to ≤240 °C. The test measures the time taken to reach a displacement of 50 mm. If the time required to reach a 50 mm displacement is <20 s, self-leveling of the mastic asphalt mixture is possible.

As listed in Table 10, RG is in a non-measurable state, and for SG and WG, the displacement reaches 50 mm within 20 s at the test temperature (240 °C), indicating that self-leveling is possible. In addition, SG exhibits excellent fluidity; self-leveling is possible up to a temperature 30 °C lower than the test temperature (210 °C).

Table 10 presents comprehensive data from the Luer fluidity test conducted across a range of temperatures (210 °C, 220 °C, 230 °C, and 240 °C) for three distinct asphalt gradations: rough (RG), soft (SG), and workability (WG). The Luer fluidity test is an essential evaluation method used to assess the flow characteristics and workability of asphalt materials at elevated temperatures, providing valuable insights for construction applications.

At the initial temperature of 210 °C, the rough gradation (RG) did not yield a measurable fluidity value. In contrast, the soft gradation (SG) demonstrated a fluidity of 17, while the workability gradation (WG) exhibited an even higher fluidity value of 25. This indicates that, at this lower temperature, the SG and WG asphalt materials are more workable and flow more readily compared to RG. Notably, SG had approximately 58% higher fluidity than WG at this temperature.

As the test temperature increased to 220 °C, both SG and WG showed improvements in fluidity, with SG achieving a fluidity value of 12 and WG reaching 21. This suggests that as the temperature rises, these materials become more fluid, making them suitable for applications requiring enhanced workability and flow characteristics. At this temperature, SG exhibited around 42% lower fluidity than WG.

At 230 °C, the trend continued, with SG and WG displaying further increases in fluidity, recording values of 8 and 18, respectively. This demonstrates the materials’ suitability for construction scenarios demanding optimal flow properties at moderately elevated temperatures. Here, SG had approximately 56% lower fluidity than WG.

The highest test temperature, 240 °C, revealed the highest levels of fluidity for both SG and WG, with values of 5 and 13, respectively. This information is vital for construction professionals who need materials that can maintain excellent workability and flow even under high-temperature conditions. At this temperature, SG had approximately 62% lower fluidity than WG.

In summary, the results from the Luer fluidity test indicate that as the temperature increases, the fluidity of the asphalt materials improves, especially for soft and workability gradations. However, the percentage differences highlight that, at each temperature, SG generally exhibited lower fluidity compared to WG, suggesting that WG is more suitable for applications requiring higher workability and flow characteristics at elevated temperatures.

### 3.3. Plastic-Deformation Resistance Evaluation

#### 3.3.1. Dynamic Stability Test

**Figure 10:**
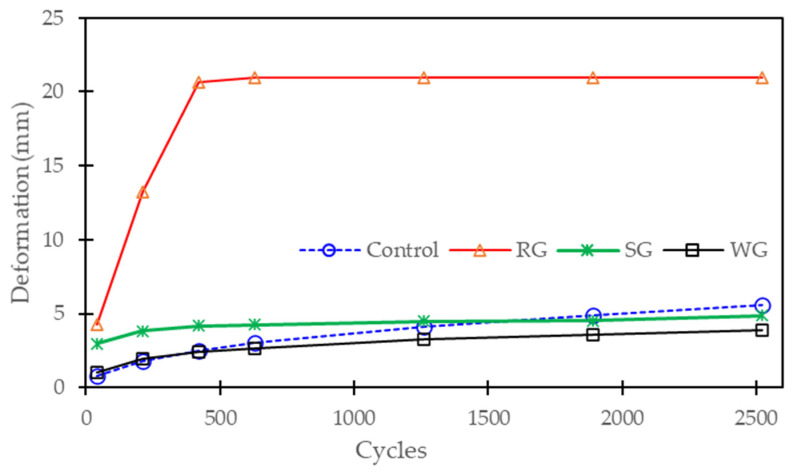
Dynamic stability test results for different particle sizes.

**Figure 11:**
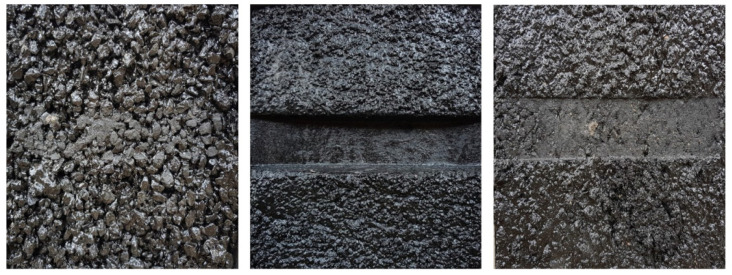
Specimens after the test (rough gradation, soft gradation, and workability gradation).

The mastic asphalt mixture uses less aggregate than typical hot mix asphalt and uses a small amount of coarse aggregate to improve workability. Thus, the intermeshing effect between aggregates cannot be expected. Therefore, the mastic asphalt mixture may be vulnerable to plastic deformation. In this study, a dynamic stability test standard [53] was performed to evaluate the plastic deformation resistance of the developed mastic asphalt mixture for expansion joints. The amount of settlement was measured by applying a wheel load to the mixture specimen (30 cm × 30 cm × 5 cm). Moreover, a 20 mm-dense asphalt mixture with good rutting resistance is used as a control group to evaluate the applicability of the mixture to the wearing course. In the test, the settlement amount of the developed mastic asphalt mixture was 40% smaller than that of the HMA. According to the dynamic stability test standard, the developed material appears to have good resistance to plastic deformation, considering that the dynamic stability of Guss asphalt was 300 cycles/mm or more, and the asphalt wearing course was 750 cycles/mm or more. Table 11 and Figure 10 summarize the dynamic stability test results, and Figure 11 shows an image of the settlement state after the test.

Table 11 and Figure 10 present the results of the dynamic stability test conducted for materials with different particle sizes, including control, rough gradation (RG), soft gradation (SG), and workability gradation (WG). The dynamic stability test is a critical assessment that helps evaluate the ability of these materials to withstand deformation and maintain structural integrity under dynamic loading conditions.

At the initial cycle (0), all materials registered low dynamic stability values, with control, RG, SG, and WG showing 0, 0, 0, and 0 cycle/mm, respectively. This signifies the materials’ initial inability to resist deformation under dynamic loading. As the test progressed to cycle 42, there was a noticeable improvement in dynamic stability for all materials, with control, RG, SG, and WG reaching 0.79, 4.30, 2.98, and 1.05 cycle/mm, respectively. This indicates an increase in the materials’ ability to withstand deformation, especially for RG. At cycle 210, there was a substantial enhancement in dynamic stability across all materials. Control, RG, SG, and WG exhibited dynamic stability values of 1.82, 13.23, 3.81, and 1.92 cycle/mm, respectively. This demonstrates that the materials, particularly RG, SG, and WG, improved significantly in their resistance to deformation under dynamic loading conditions.

The trend continued at cycles 420, 630, 1260, 1890, and 2520, with dynamic stability values consistently increasing for all materials. This signifies their ability to withstand deformation and maintain structural integrity over an extended duration of dynamic loading. At cycle 2520, control, RG, SG, and WG achieved dynamic stability values of 5.55, 20.97, 4.85, and 3.88 cycle/mm, respectively, showcasing their improved performance in resisting deformation.

The rate of deformation (RD) remained relatively consistent for all materials throughout the test, indicating that the materials responded similarly to the applied deformation rate. The dynamic stability (DS) values clearly illustrate that, among the materials tested, SG exhibited the highest dynamic stability at cycle 2520, followed by WG, RG, and control.

Firstly, it is evident that RG consistently outperforms the other mixtures across all tested cycles. At 1260 cycles, RG achieves a remarkable 20.97 cycles/mm, indicating a 413% improvement compared to the control’s 4.09 cycles/mm. This substantial difference underscores the advantage of employing RG in asphalt mixtures for enhanced structural stability. SG also demonstrates a significant performance boost compared to the control. At the same 1260-cycle mark, SG exhibits a dynamic stability value of 4.48 cycles/mm, marking a 9% increase over the control. While this percentage difference may appear smaller than RG’s, it still signifies an important enhancement in the mixture’s ability to resist deformation. In contrast, WG, while offering improvements over the control, falls behind RG and SG in terms of dynamic stability. At 1260 cycles, WG shows a value of 3.26 cycles/mm, indicating a 20% improvement over the control. While this improvement is notable, the lower dynamic stability compared to RG and SG suggests that the particle size distribution and gradation of WG may not be as conducive to resisting deformation.

These percentage comparisons underscore the critical role of particle size distribution and gradation in determining the dynamic stability of asphalt mixtures. RG emerges as the standout performer, offering a substantial 413% improvement over the control, while SG and WG also deliver significant enhancements of 9% and 20%, respectively. These findings can guide the selection of asphalt mixtures for construction projects, ensuring the durability and longevity of the pavement structure.

In conclusion, the dynamic stability test results highlight the materials’ improved resistance to deformation under dynamic loading conditions as the test cycles progressed. Among the materials, soft gradation (SG) demonstrated the highest dynamic stability, making it a promising choice for applications where resistance to deformation is crucial.

#### 3.3.2. Hamburg Wheel Tracking Test

The Hamburg flexural tracking test, a comprehensive evaluation method for assessing moisture susceptibility, stripping resistance, and plastic deformation characteristics of asphalt mixtures, provides crucial insights into the performance of various asphalt formulations. As per the guidelines outlined in AASHTO T324 (American Association of State Highway and Transportation Officials, 2022) [54], this test involves subjecting an asphalt mixture specimen to repeated wheel load applications while immersed in water at an elevated temperature of 60 °C. In the context of this study, the Hamburg wheel tracking test served as a pivotal tool for comparing the newly proposed asphalt mixture with the control group, which comprised a conventional 20 mm-dense asphalt mixture. The objective was to assess the asphalt mixture containing workability gradation (WG) particles that satisfied the criteria established by both the Luer fluidity test and the dynamic stability test.

**Figure 12:**
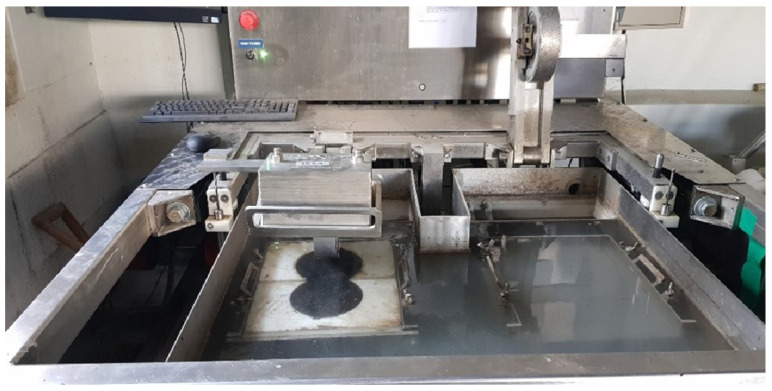
Hamburg wheel tracking setup.

Figure 12 illustrates the setup, where the specimen mixture is mounted onto the Hamburg wheel tracking equipment to undergo testing. The results obtained for the WG material are particularly noteworthy, revealing a settlement amount that is 40% smaller than that observed for the control group. This substantial reduction in settlement holds significant implications for the durability and longevity of the pavement structure. Notably, in the case of the control group, the deformation amount increased rapidly after approximately 8000 cycles, signifying its vulnerability to distress under high-stress conditions. In stark contrast, the developed WG material exhibited a relatively gentle slope, suggesting its superior resistance to deformation over an extended service life.

**Figure 13:**
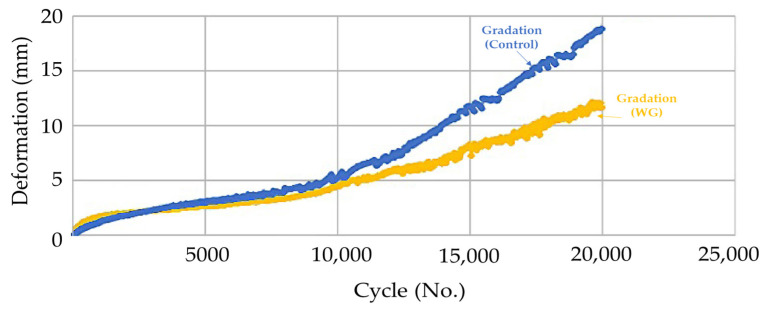
Hamburg wheel tracking test results.

**Figure 14:**
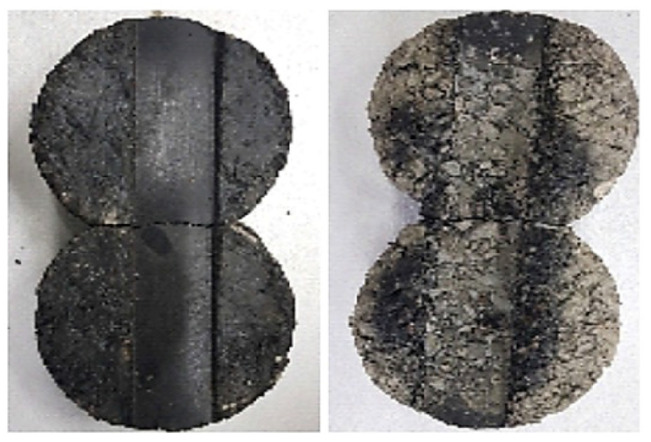
Specimens after the Hamburg wheel tracking test: workability gradation (**left**) and control (**right**).

Figure 13 and Figure 14 provide visual evidence of the surface conditions of the specimen mixture after undergoing the Hamburg wheel tracking test. These images vividly depict the stark contrast in performance between the control group and the developed material (WG). In the control group, severe stripping is readily noticeable, indicating a susceptibility to moisture-induced damage and plastic deformation. However, for the developed WG material, instances of stripping are conspicuously absent. This observation reinforces the conclusion that the developed material boasts exceptional resistance to plastic deformation and moisture, making it a promising choice for asphalt pavement construction and maintenance. The substantial 40% reduction in settlement, coupled with the visual evidence of superior resistance, underscores the significance of incorporating WG particles into asphalt mixtures to enhance their long-term performance and mitigate distress mechanisms.

The performance gains achieved through the incorporation of self-leveling SG and WG gradations are indeed remarkable. These mixtures, designed to flow more freely, have exhibited dynamic stability values that surpass those of high-density mixtures renowned for their excellent flow resistance by over two-fold. This substantial improvement underscores the potential of these gradations to enhance the overall structural integrity of asphalt pavements.

Furthermore, the results of the Hamburg wheel tracking test, a comprehensive assessment encompassing moisture susceptibility, stripping resistance, and plastic deformation characteristics, reveal the impressive capabilities of the developed material with WG gradation. It notably enhances plastic deformation resistance by reducing settlement by more than 40% when compared to the control group. This reduction in settlement is a critical finding, as it directly translates into increased pavement durability and longevity.

Notably, visual evaluations of specimen conditions after testing indicate minimal to no stripping in the developed material, affirming its robust performance and suitability for applications such as expansion joint materials. However, it is important to acknowledge that these results are derived from controlled laboratory testing conditions, and real-world performance may vary. Therefore, it remains imperative to conduct extensive long-term performance evaluations through construction and field tests to ascertain how these promising materials fare under actual driving conditions. These ongoing efforts will provide valuable insights into the practical applicability and sustained benefits of the developed asphalt mixtures in real-world road infrastructure.

### 3.4. Discussions 

The findings of this study contribute to the existing body of knowledge on expansion joint materials and their potential for improving the durability of bridge expansion joints. Our research demonstrates that the selection of styrene–butadiene–styrene (SBS) as a modifier for rubber-modified asphalt significantly enhances the elasticity, adhesion, and workability of the asphalt binder, aligning with previous studies. The polymer-modified rubber asphalt binder developed in this study exhibits an impressive elastic recovery rate of 61.1–66.1%, surpassing the performance of commercial products. The reduced viscosity, 15–21% lower than existing commercial products, further emphasizes the superior workability achieved through our novel approach. This aligns with the objectives of our research, which aimed to enhance the constructability and workability of mastic asphalt mixtures used in expansion joints.

Furthermore, the improved performance of the mastic asphalt mixture as a filling material for expansion joints, particularly in terms of plastic deformation resistance, is consistent with the objectives of our research. The settlement amount, reduced by 40% compared to hot mix asphalt for the surface layer, highlights the potential of our developed material to address durability challenges in existing asphalt plug joints. Moisture and stripping resistances were also notably improved, underscoring the multifaceted benefits of our approach.

The success of this study in developing a novel polymer-modified rubber asphalt-based expansion joint filling material with enhanced properties contributes to addressing the critical issue of premature expansion joint failure. Our findings provide valuable insights into the construction and maintenance of bridges, ultimately improving their service life and reducing associated repair costs. Nevertheless, it is essential to acknowledge that while laboratory testing has yielded promising results, real-world performance may be influenced by various external factors, such as traffic loads and environmental conditions. Therefore, future work should focus on conducting comprehensive field trials and long-term monitoring to validate the long-term performance of the developed materials in practical applications.

## 4. Conclusions

In this comprehensive study, a pioneering expansion joint filling material was meticulously developed with the primary goal of enhancing the durability of expansion joints in road infrastructure. Through a series of rigorous tests and evaluations, several key conclusions have emerged:

- The selection of SBS as a modifier for the material proved to be judicious. SBS, renowned for its outstanding elasticity, adhesion, and applicability, played a pivotal role in crafting a polymer-modified rubber asphalt binder.
- The novel asphalt binder exhibited remarkable elastic recovery rates, surpassing those of existing commercial products by a significant margin, falling within the impressive range of 61.1–66.1%.
- The viscosity of the developed asphalt binder was notably lower, by 15–21%, compared to its commercial counterparts, signifying superior workability.
- The storage stability tests revealed the superiority of this material, with higher softening points and less variation between sections. Moreover, the Frequency Sweep Test results revealed variations in stiffness among the tested mixtures, with the modified binder exhibiting greater stiffness compared to the control counterpart.
- The study resulted in the formulation of a mastic asphalt mixture for expansion joint filling, featuring the innovative use of a cement–concrete mixing particle size—an unconventional choice aimed at enhancing workability.
- The workability evaluation demonstrated that self-leveling can be effectively achieved at a moderate temperature of 210 °C, effectively overcoming the limitations associated with traditional mastic asphalt mixtures, which often suffer from poor constructability and workability.
- The dynamic stability test and Hamburg wheel tracking test outcomes presented compelling evidence of this material’s durability, with deformation levels consistently measuring 40% less than those observed in conventional 20 mm-dense hot mix asphalt (HMA).
- These findings underscore the material’s exceptional resistance to plastic deformation, further validated by the near absence of surface stripping following the Hamburg wheel tracking test. These results collectively attest to the material’s outstanding moisture and stripping resistance. The newly proposed filling material not only demonstrated remarkable workability and durability but also holds the promise of addressing longstanding challenges in expansion joint construction.
- Looking ahead, the focus will shift towards substantiating the long-term performance of these pioneering materials through comprehensive construction and pavement acceleration tests, providing invaluable insights into their practical viability and sustained benefits within real-world road infrastructure.

## Figures and Tables

**Table 1:** Physical properties of elastic force-imparting polymers.

Property	SBS	SBR/BR	CRM
Vulcanization process	Not necessary	Necessary	Not necessary
Softening point	High		Low
Elastic resilience	High		
Process suitability	Excellent	Equipment/process adjustment required	Excellent
Asphalt	Excellent	Moderate	

**Table 2:** Physical properties of asphalt binder materials with different styrene–butadiene–styrene structures.

Property	3-Linear Type	3-Linear Type	3-Radial Type
Softening point, °C	108.3	92.4	114.2
Ductility, cm (at 25 °C)	110	140	75
Penetration, dm (at 25 °C, 100 g, 5 s)	19.6	23.4	13.5
Viscosity, cPs (at 135 °C)	6700	5600	11,700
Elastic recovery, % (at 25 °C)	95	87	94

**Table 3:** Physical properties of petroleum resins.

Property	Type A	Type B	Type C
Grade	G140	G120	G1100
Softening point, °C	145	120	100
Compatibility with asphalt	Excellent	Excellent	Moderate

**Table 4:** Physical properties of the asphalt binder containing petroleum resin.

Property	Type A	Type B	Type C
Grade	G140	G120	G1100
Softening point, °C	98.3	92.7	85.4
Stripping resistance, kgf/cm (at 90 °C)	2.21	2.32	2.22
Phase segregation, Δ°C (at 163 °C, 48 h)	2.2	2.3	13.1

**Table 5:** The mixed proportion of the filling asphalt plug joint material for the developed expansion joint.

Division	Grade	Composition Ratio (%)	
		Type 1	Type 2
Asphalt	PG 64-22	75	75
SBS	101	10	6
	104	0	4
Petroleum resin	G140	10	10
Oil	GS150	5	5
Mineral	CaCO_3_	0	0

SBS, styrene–butadiene–styrene.

**Table 6:** Physical properties of the first developed expansion joint filling material.

ASTM D6297	ASTM	Unit	Spec.	Control	Type 1	Type 2
Softening point	D36	°C	83	88.5	108.3	103.4
Ductility (at 25 °C)	D113	mm	>400	440	760	880
Recommended application heating	-	-	182–199	satisfied	satisfied	satisfied
Penetration (at 25 °C, 100 g, 5 s)	D5	dm	-	23.8	27.8	33.9
Elastic recovery (at 25 °C)	-	%	-	77	94	88
Viscosity (at 135 °C)	D446	cPs	-	21355	6691	5675

ASTM, American Society for Testing and Material.

**Table 7:** Physical properties of the final developed expansion joint filling material.

ASTM D6297	ASTM	Unit	Spec.	Control	Type 3	Type 4
Softening point	D36 [44]	%	83	88.5	102.5	104.5
Tensile adhesion	D5329 [45]	%	>700	722	823	785
Ductility (at 25 °C)	D113 [46]	mm	>400	440	760	570
Cone penetration (at 25 °C, 100 g, 5 s)	D5329 [45]	unit	<75	16.3	23.3	18.1
Cone penetration (at −18 °C, 200 g, 60 s)	D6591 [47]	unit	>10	11.1	12.1	10.6
Flow (at 60 °C, 5 h)	D5329 [45]	mm	<3.0	0.55	0.87	0.51
Resiliency (at 25 °C)	D5329 [45]	%	40–70	44.5	66.1	61.1
Asphalt compatibility	D5329 [45]	-	pass	-	pass	pass
Recommended application heating temperature range	-	-	182–199	satisfied	satisfied	satisfied
bond 3 cycles (at −7 °C, 100% elongation)	D5329 [45]	-	pass	-	pass	pass
Flexibility (at 23 °C)		-	pass	-	pass	pass
Penetration (max. at 25 °C, 100 g, 5 s)	D5 [48]	dm		23.8	34.4	20.4
Elastic recovery (at 25 °C)		%		77	100	97
Viscosity (at 135 °C)	D446 [49]	cPs		21355	3200	4500

ASTM, American Society for Testing and Material.

**Table 8:** Quality of the mastic asphalt binder.

Division	Spec.	Test Result
Softening point, °C	>83	102.5
Tensile adhesion, %	>700	823
Ductility, mm (at 25 °C)	>400	760
Penetration, dm (at 25 °C)	-	34.4
Elastic recovery, % (at 25 °C)	-	100
Viscosity, cPs (at 25 °C)	-	3200

**Table 9:** Storage stability test results based on the difference in the softening point.

Softening Point (°C)	Control	Type 3	Type 4
Unaged sample	62.6	65.2	64.8
Top section	63.5	65.0	64.9
Bottom section	62.9	65.1	64.4
Softening point difference (°C)	0.9	0.2	0.5

**Table 10:** Results of the Luer fluidity test.

Temp. (℃)	RG	SG	WG
210	Not measured	17	25
220		12	21
230		8	18
240		5	13

RG, rough gradation; SG, soft gradation; WG, workability gradation.

**Table 11:** Dynamic stability test results for different particle sizes.

Cycle	Control	RG	SG	WG
0	0	0	0	0
42	0.79	4.30	2.98	1.05
210	1.82	13.23	3.81	1.92
420	2.49	20.69	4.16	2.41
630	3.00	20.96	4.24	2.67
1260	4.09	20.97	4.48	3.26
1890	4.88	20.97	4.53	3.60
2520	5.55	20.97	4.85	3.88
Rate of Deformation (RD) (mm/min)	0.04	0	0.02	0.02
Dynamic Stability (DS) (cycle/mm)	940	0	1969	2250
Deformation (mm)	5.55	20.97	4.85	3.88

RG, rough gradation; SG, soft gradation; WG, workability gradation.

## Data Availability

The data presented in this study are available on request from the corresponding author.
